# Comsolic solution of an elliptic cylindrical compressible fluid flow

**DOI:** 10.1038/s41598-021-99138-7

**Published:** 2021-10-08

**Authors:** Azad Hussain, Ali Hassan, Qasem Al Mdallal, Hijaz Ahmad, El-Sayed M. Sherif, Aysha Rehman, Mubashar Arshad

**Affiliations:** 1 Department of Mathematics, University of Gujrat, Gujrat, 50700 Pakistan; 2 Department of Mathematical Sciences, UAE University, 15551, Al Ain, UAE; 3 Section of Mathematics, International Telematic University Uninettuno, Corso Vittorio Emanuele II, 39, 00186 Roma, Italy; 4 Department of Mechanical Engineering, College of Engineering, King Saud University, P.O. Box 800, Al-Riyadh, 11421 Saudi Arabia

**Keywords:** Energy science and technology, Mathematics and computing, Nanoscience and technology

## Abstract

In this article, the primary focus is to investigate the heat transfer effects with viscous compressible laminar flow in the permeable elliptic cylinder. The Reynolds number is kept 100 for flow to be laminar. The physics of heat transfer is selected to be coupled with the laminar flow. The results for particular step-size time for Velocity distribution, pressure profile, temperature profile, isothermal temperature contours, and drag coefficient have been analyzed. Mesh has been generated through COMSOL, mesh entities have been elaborated statistically. The maximum and minimum velocity profile is observed at the elliptical cylinder’s walls and upper, lower boundary respectively. The maximum velocity observed is 2.22 m/s. Pressure profile around elliptic corners is found maximum, distinct patterns are observed even under the influence of applied heat. Temperature is observed maximum at walls but it gradually increases as moving from the upper boundary towards the lower boundary. The isothermal contour patterns are observed maximum near the walls, drag coefficient of gradual decrease is observed. COMSOL multi-physics is utilized for mathematical modeling of problems and the Backward-Differentiation-Formula has been exploited to handle problems numerically. The results will help greatly to understand the characterizations of viscous fluids and in industries like air furnaces and automobile cooling systems.

## Introduction

Numerous applications that have been utilized today either handmade or mechanically constructed have a distinguished component known as Heat transfer. The application heat transfer counts on fluid’s thermal conductivity. Fluid heat transfer is established by liquidizing nano-sized particles, which are nano-meter-scale of measurement (1–100) nm, in the customary transfer of heat. The metallic and non-metallic nanofluid particle shapes are formed by semiconductors, metals, and nitride ceramics. These nanofluids are considered strong conductive mediums. In the base fluids, when a nano-sized particle is distributed it automatically enhances the thermal conductivity of nanofluid^1^.

Nadeem et al.^2^ explored three-dimensional hybrid nanofluid at stagnation point to analyze heat transfer coefficient past a circular cylinder. Nadeem et al.^3^ discussed the boundary layer flow of second-grade fluid with heat transfer in a cylinder. Turkyilmazoglu^4^ introduced the purely analytical solution of compressible boundary layer flow due to a porous disk along with heat transfer. Asghar et al.^5^ have analyzed the Lie group for flow with heat transfer over a stretching rotating disk. Turkyilmazoglu^6^ has discussed the effects of uniform radial electric field on MHD heat and fluid flow due to rotating disks.

The nanofluids are generated by distributing nanometer-sized particles in a base liquid. These fluids are significantly utilized in industries to enhance the heat and mass transfer rates. Industries like, pharmaceutical formulations, mixing of food, painting, blood flow, liquid flow, alloy metal flow, polymer solutions, mercury mix flow, and nuclear fuel. Thermal conductivity plays a vital role in increasing the heat transfer coefficient of nanofluids. The hybrid nanofluids have greater thermal conductivity but still, there are chances that the nanofluid two-phase model provides good results^7^.

Rehman et al.^8^ has explored the molecular theory of liquid-originated nanofluid with variable properties under physical aspects of the convective and radiative effects. Hussain et al.^9^ has introduced a model for convectively heated surface near a stagnation point for Carreau-Yasuda nanofluid. Mebarek-Oudina et al.^10^ have studied special porous enclosures for convective heat transfer of hybrid nanofluid. Rehman et al.^11^ have analyzed a flexible Riga sheet for pseudo-plastic nano liquid flow with assisting and opposing stagnation points. Numerous scientists have examined nanofluids to study different physical aspects, the hybrid and nanofluids have been utilized^12–15^, referred for study purposes.

The boundary layer flow for compressible fluid over the years has been investigated and utilized to incredibly increase the heat transfer coefficient and reduce the occurring fiction in mechanical procedures. The quest of achieving more suitable results through the examination of compressible fluid’s thermal boundary on the surface of an aircraft either it behaves in a laminar or turbulent manner has urged scholars to examine it in depth. Studies suggest that turbulent flow has much greater rates of skin profiles and heat transfer when compared to laminar flow, any numerical technique is worth investigating to stabilizing the boundary layer of compressible viscous flow^16^.

Vajravelu^17^ has discussed the viscous flow over a non-linearly stretching sheet. Lin and Rubin^18^ have explored the three-dimensional supersonic viscous flow over a cone at incidence. Malik and Spall^19^ studied the stability of compressible fluid over axisymmetric bodies. Cantwell^20^ has produced marvelous work “Fundamentals of compressible flow” to study compressible fluids. Kumar and Sivaraj^21^ have discussed heat and mass transfer in MHD viscoelastic fluid for both cone and plate. Houda et al.^22^ have introduced the CDF COMSOL model for simulating the urban complex flow. Fröhlingsdorf^23^ has discussed the numerical investigation of compressible flow and energy separation in the Ranque-Hilsch vortex tube. Many researchers explored the useful studies in articles^24–31^. Bhandari et al.^32^ have explored time-dependent ferrofluid past a circular cylinder in the presence of stationary magnetic-filed.

The purpose of this article is to investigate the laminar flow of viscous compressible time-dependent fluid for an elliptic cylinder. The laminar flow is coupled with the heat transfer feature. The mathematical modeling of elliptic cylindrical flow is done in COMSOL. The outcomes are discussed for velocity, pressure temperature, and isothermal temperature distribution. The mesh created through COMSOL has been described with comprised constitutes. The thermophysical properties of water-based fluid that have been utilized are elaborated. BDF (backward differentiation formula) also known as the Backward-Euler-method technique is used to numerically handle the model in COMSOL.

## A mathematical formulation of the problem

The sketch of the flow region is given below. First of all, a rectangular geometry is drawn in the COMSOL with specifics as $2.0$ m in width and $0.4$ m in height with coordinates position as $\left( {{\text{r}},{\text{z}}} \right) \left( {0,0} \right)$. In the two-dimensional plots, the vertical and horizontal axis present’s the diameter of the elliptic cylinder and z-direction respectively. An ellipse is drawn at coordinates position $\left( {0.3,0.2} \right)$, with $\left( {0.15,0.05} \right)$ being a-semi and b-semi axis. The difference of both geometrical shapes is taken through booleans and partitions command and then through building all command final geometry is obtained. The heat transfer in fluids physics is added in COMSOL, in the initial values the temperature is taken $200$ K, temperature 1 (taken $298$ K on the upper boundary), and temperature 2 (taken $300.5$ K on the lower boundary) are added under heat transfer segment.

In the next step, properties of fluid have been added from material properties. The thermal conductivity, fluid density, and others properties are added. In COMSOL, ${\text{u}},{\text{v}},{\text{w}}$ are taken as dependent variables for velocity components in respective directions, and ${\text{T}}$ is considered a dependent variable for temperature.

The model governing Eqs. (1–3) comes up by default method in COMSOL, as we model the geometry. The momentum equation is given as

1 $$\uprho \frac{{\partial {\text{u}}}}{{\partial {\text{t}}}} +\uprho \left( {{\text{u}} \cdot \nabla } \right){\text{u}} = - \nabla \left( {{\text{pI}}} \right) + \nabla\upgamma + {\text{F}}_{{\text{b}}} ,$$

where ${\uprho }$ is the fluid density, u being fluid velocity, ${\upgamma }$ is useful relation in numerical proceedings and ${\text{F}}_{{\text{b}}}$ denotes body forces. The equation of continuity is

2 $${ }\frac{{\partial {\uprho }}}{{\partial {\text{t}}}} + \nabla \cdot \left( {{\rho u}} \right) = 0,$$

as the model is coupled with the heat transfer, the governing equation for heat transfer is given as:

3 $${\text{d}}_{{\text{z}}} {\rho c}_{{\text{p}}} \left( {\frac{{\partial {\text{T}}}}{{\partial {\text{t}}}}} \right) + {\text{d}}_{{\text{z}}} {\rho c}_{{\text{p}}} \left( {{\text{u}}\nabla {\text{T}}} \right) + \nabla {\text{q}} = {\text{d}}_{{\text{z}}} {\text{Q}} + {\text{q}}_{0} + {\text{d}}_{{\text{z}}} {\text{Q}}_{{\text{p}}} + {\text{d}}_{{\text{z}}} {\text{Q}}_{{\text{vd }}} .$$

${\text{d}}_{{\text{z}}}$ represents the thickness of geometry, ${\text{c}}_{{\text{p}}}$ presents constant pressure heat capacity, ${\text{T}}$ denotes absolute temperature, q presents heat flux vector, Q is heat source other than viscous dissipation, ${\text{Q}}_{{\text{p}}}$ is heat transfer at constant pressure and ${\text{Q}}_{{\text{vd }}}$ is the heat source via viscous dissipation.

Where the relation consists of the following expressions:

4 $${\text{Q}} = 0,\;{\text{q}}_{0} = \frac{{\text{q}}}{{{\text{A}}_{{\text{s}}} \Delta {\text{T}}}},\;{\text{Q}}_{{\text{p}}} = {\upalpha }_{{\text{p}}} {\text{T}}\left( {\frac{{\partial {\text{P}}}}{{\partial {\text{t}}}} + {\text{u}}\nabla {\text{P}}} \right),\;{\text{and}}\;{\text{Q}}_{{{\text{vd}}}} = {\uptau } \cdot \nabla {\text{u}},$$

where $\Delta {\text{T}}$ is a temperature difference, ${\text{A}}_{{\text{s}}}$ is area commuted by default through the mesh, ${\upalpha }_{{\text{p}}}$ is the rate of change pressure with respect to absolute temperature, ${\text{q}}_{0}$ is heat flux at a certain time and ${\uptau }$ is viscous stress tensor.

5 $${\text{q}} = - {\text{k}}\nabla {\text{T}},\;{\upalpha }_{{\text{p}}} = \frac{ - 1}{{\uprho }}\frac{{\partial {\text{P}}}}{{\partial {\text{T}}}}\;{\text{and}}\;{\text{also}}\;{\uptau } = - {\text{pI}} + {\mu A}_{1} ,\;{\uptau }_{{{\text{race}}}} \left( {{\uptau } \cdot \nabla {\text{u}}} \right) = {\uptau } \cdot \nabla {\text{u}},$$

Here, p represents pressure, I is the identity matrix, ${\text{A}}_{1}$ is an expression mentioned below and k denotes fluid thermal conductivity.

6 $$\begin{aligned} & {\text{A}}_{1} = \left( {\left( {\nabla {\text{u}}} \right) + \left( {\nabla {\text{u}}^{{\text{T}}} } \right)} \right),\;\Delta {\text{T}} = {\text{T}}_{1} - {\text{T}}_{2} { },\;{\text{A}}_{{\text{s}}} = 0.8\;{\text{m}}^{2} { }, \\ & {\text{d}}_{{\text{z}}} = 1\;{\text{m }}\left( {{\text{thickness}}} \right), \\ & {\upgamma } = {\upmu }\left( {\left( {\nabla {\text{u}}} \right) + \left( {\nabla {\text{u}}^{{\text{T}}} } \right)} \right) - \frac{2}{3}{\upmu }\left( {\nabla {\text{u}}} \right){\text{I}}. \\ \end{aligned}$$

Here, ${\upgamma }$ is a factor contributing to solving governing Eq. (1) and ${\upmu }$ denotes fluid’s kinematic viscosity.

The body forces are neglected. The momentum Eqs. (8–10) and continuity Eq. (7) are utilized from^32^. Equations (14, 15) are utilized from^33^. The continuity, momentum, and heat transfer at constant pressure will reduce to:

7 $$\frac{{\partial {\uprho }}}{{\partial {\text{t}}}} + {\uprho }\left( {\frac{{\partial {\text{u}}_{{\text{r}}} }}{{\partial {\text{r}}}} + \frac{{{\text{u}}_{{\text{r}}} }}{{\text{r}}} + \frac{{\partial {\text{u}}_{{\text{z}}} }}{{\partial {\text{z}}}}} \right) = 0,$$

8 $$\left[ {\frac{{\partial {\text{u}}_{{\text{r}}} }}{{\partial {\text{t}}}} + {\text{u}}_{{\text{r}}} \frac{{\partial {\text{u}}_{{\text{r}}} }}{{\partial {\text{r}}}} + {\text{u}}_{{\text{z}}} \frac{{\partial {\text{u}}_{{\text{r}}} }}{{\partial {\text{z}}}} - \frac{{{\text{u}}_{{\uptheta }}^{2} }}{{\text{r}}}} \right] = - \frac{ - 1}{{\uprho }}\frac{{\partial {\text{P}}}}{{\partial {\text{r}}}} + {\upnu }\left[ {2\frac{{\partial^{2} {\text{u}}_{{\text{r}}} }}{{\partial {\text{r}}^{2} }} + \frac{{\partial^{2} {\text{u}}_{{\text{r}}} }}{{\partial {\text{r}}\partial {\text{z}}}} + \frac{1}{3}\frac{{\partial^{2} {\text{u}}_{{\text{r}}} }}{{\partial {\text{z}}^{2} }}} \right],$$

9 $$\left[ {\frac{{\partial u_{\theta } }}{\partial t} + u_{r} \frac{{\partial u_{\theta } }}{\partial r} + u_{z} \frac{{\partial u_{\theta } }}{\partial z} + \frac{{u_{\theta } u_{r} }}{r}} \right] = \nu \left[ {\frac{1}{3}\frac{{\partial^{2} {\text{u}}_{{\uptheta }} }}{{\partial {\text{r}}^{2} }} - \frac{1}{{\text{r}}}\left( {\frac{{\partial {\text{u}}_{{\uptheta }} }}{{\partial {\text{r}}}}} \right) + \frac{1}{3}\frac{{\partial^{2} {\text{u}}_{{\uptheta }} }}{{\partial {\text{z}}^{2} }}} \right],$$

10 $$\left[ {\frac{{\partial {\text{u}}_{{\text{z}}} }}{{\partial {\text{t}}}} + {\text{u}}_{{\text{r}}} \frac{{\partial {\text{u}}_{{\text{z}}} }}{{\partial {\text{r}}}} + {\text{v}}_{{\text{z}}} \frac{{\partial {\text{u}}_{{\text{z}}} }}{{\partial {\text{z}}}}} \right] = - \frac{ - 1}{{\uprho }}\frac{{\partial {\text{P}}}}{{\partial {\text{z}}}} + {\upnu }\left[ {\frac{1}{3}\frac{{\partial^{2} {\text{u}}_{{\text{z}}} }}{{\partial {\text{r}}^{2} }} + \frac{{\partial^{2} {\text{u}}_{{\text{r}}} }}{{\partial {\text{z}}\partial {\text{r}}}} + 2\frac{{\partial^{2} {\text{u}}_{{\text{z}}} }}{{\partial {\text{z}}^{2} }}} \right],$$

Here, in Eqs. (7) to (10) the ${\text{u}}_{{\text{r}}}$, ${\text{u}}_{{\uptheta }}$, ${\text{u}}_{{\text{z}}}$ are velocity components in respective r, ${\uptheta }$, and z-direction, ${\upnu }$ denotes dynamic viscosity and ${\uprho }$ fluid’s density. The general heat equation at variable pressure:

11 $${\rho c}_{{\text{p}}} \left( {\frac{{\partial {\text{T}}}}{{\partial {\text{t}}}} + {\text{T}}\nabla {\text{u}}} \right) - {\text{k}}\nabla^{2} {\text{T}} = \frac{{\text{q}}}{{{\text{A}}_{{\text{s}}} \Delta {\text{T}}}} - \frac{1}{{\uprho }}\frac{{\partial {\text{p}}}}{{\partial {\text{t}}}}\frac{{\partial {\text{t}}}}{{\partial {\text{T}}}}\left( {\frac{{\partial {\text{p}}}}{{\partial {\text{t}}}} - {\text{p}}\nabla {\text{u}}} \right) + \left( { - {\text{pI}} + {\mu A}_{1} } \right)\nabla {\text{u}},$$

also at constant pressure:

12 $${\rho c}_{{\text{p}}} \left( {\frac{{\partial {\text{T}}}}{{\partial {\text{t}}}} + {\text{T}}\nabla {\text{u}}} \right) - {\text{k}}\nabla^{2} {\text{T}} = \frac{{\text{q}}}{{{\text{A}}_{{\text{s}}} \Delta {\text{T}}}} + {\mu A}_{1} \left( {\nabla {\text{u}}} \right),$$

where ${\uprho }$ denotes density, t describes time,$\nabla {\text{T}}$ denotes temperature gradient, $\Delta {\text{T}}$ describes temperature difference, ${\text{A}}_{{\text{s}}}$ describes the area of geometry, ${\text{A}}_{1}$ is an above-defined expression, ${\upmu }$ is kinematic viscosity, k is thermal conductivity, T presents absolute temperature, ${\text{c}}_{{\text{p}}}$ presents constant pressure heat capacity and q denotes heat flux vector.

The similarity assembly for the heat equation is as follow:

13 $${\text{T}}\left( {{\text{t}},{\text{r}},{\text{z}}} \right) - {\text{T}}_{\infty } = \left( {{\text{T}}_{{\text{w}}} - {\text{T}}_{\infty } } \right){\uptheta },\;{\text{T}}_{{\text{w}}} - {\text{T}}_{\infty } = \left( {{\text{T}}_{0} - {\text{T}}_{\infty } } \right)\frac{{\text{x}}}{{\text{L}}}\left( {1 - {\text{st}}^{*} } \right)^{ - 2} ,\;{\text{t}}^{*} = \left( {\Omega \sin \upalpha ^{*} } \right){\text{t}},$$

where

14 $$\upeta = \frac{{{\text{z }}\left( {\Omega \sin \upalpha ^{*} } \right)^{0.5} }}{{{\text{v}}^{0.5} \left( {1 - {\text{st}}\;\Omega \sin \upalpha ^{*} } \right)^{0.5} }},\;{\text{G}} = \left( {\Omega \sin \upalpha ^{*} } \right)^{ - 1} \left( {1 - {\text{st}}\;\Omega \sin \upalpha ^{*} } \right).$$

And ${\nu }$ is the kinematic viscosity, ${\text{T}}_{{\text{w}}}$ denotes wall temperature and ${\text{T}}_{\infty }$ represents free stream velocity. The above-mentioned similarity has been utilized to transform the heat equation at constant pressure (12) to obtain the desired heat transfer equation for compressible fluid flow (15).

15 $$\begin{aligned} \Pr \left( {\uptheta^{\prime \prime } } \right) & = \upnu \left( {\frac{1}{2}\uptheta^{\prime } \upeta + 2\uptheta {\text{s}}} \right) - {\text{pr}}\frac{{{\text{G}}\upnu }}{{{\text{k}}\left( {{\text{T}}_{{\text{w}}} - {\text{T}}_{\infty } } \right)}}\left[ {\frac{{{\text{q}}_{0} }}{{{\text{A}}_{{\text{s}}} \Delta {\text{T}}}}} \right. \\ & \quad + \left\{ {\upmu \left( {\frac{{\partial^{3} }}{{\partial {\text{r}}^{3} }}\left( {\frac{4}{3}{\text{u}}_{{\text{r}}} + {\text{u}}_{\uptheta } + {\text{u}}_{{\text{z}}} } \right) + \frac{1}{3}\upmu \frac{{\partial^{2} }}{{\partial {\text{r}}^{2} }}\left( {\frac{{{\text{u}}_{\uptheta } }}{{\text{r}}} + \frac{{\partial {\text{u}}_{{\text{r}}} }}{{\partial {\text{z}}}}} \right)} \right.} \right. \\ & \quad - \frac{{\partial {\text{u}}_{\uptheta } }}{{\partial {\text{z}}}}\left( {{\text{p}} - \frac{4}{3}\upmu - \frac{1}{3}\frac{{\partial {\text{u}}_{{\text{z}}} }}{{\partial {\text{z}}}}\frac{{\partial {\text{u}}_{\uptheta } }}{{\partial {\text{z}}}}} \right) - \frac{{\partial {\text{u}}_{\uptheta } }}{{\partial {\text{z}}}}\left( {\frac{1}{3}\frac{{\partial {\text{u}}_{{\text{r}}} }}{{\partial {\text{z}}}} - \frac{{{\text{u}}_{\uptheta } }}{{\text{r}}}} \right) \\ & \quad \left. {\left. {\left. { - \frac{{{\text{u}}_{{\text{r}}} }}{{\text{r}}}\left( {{\text{p}} - \frac{4}{3}\upmu \frac{{{\text{u}}_{{\text{r}}} }}{{\text{r}}}} \right) - \upmu \frac{{{\text{u}}_{\uptheta } }}{{\text{r}}}\left( {\frac{1}{3}\frac{{\partial {\text{u}}_{\uptheta } }}{{\partial {\text{r}}}} - \frac{{{\text{u}}_{\uptheta } }}{{\text{r}}}} \right) - {\text{p}}\frac{{\partial {\text{u}}_{{\text{r}}} }}{{\partial {\text{r}}}}} \right)} \right\}} \right] \\ \end{aligned}$$

In Eq. (15), ${\text{Pr}} = \frac{{\text{k}}_{\text{f}}}{{p}{C}}_{{\text{p}}}$ is Prandtl number nondimensional, ${\upeta }$ is the dimensionaless parameter, ${\upnu }$ denotes fluid’s dynamic viscosity, ${\upmu }$ is kinematic viscosity, ${\text{u}}_{{\text{r}}} ,{\text{ u}}_{{\uptheta }} ,{\text{ u}}_{{\text{z}}}$ are the velocity components, ${\text{T}}_{{\text{w}}}$ denotes wall temperature, ${\text{T}}_{\infty }$ represents free stream velocity and $\Delta {\text{T}}$ denotes temperature difference, k denotes thermal conductivity, ${\text{A}}_{{\text{s}}}$ is an area of geometry, ${\uptheta }$ is non-dimensional temperature, ${\text{q}}_{0}$ is heat flux at a certain time ${\text{G}, Re} = \frac{{\text{G}}_{p}} {v}$ is the expression defined above and Re is Reynolds number respectively.

**Figure 1:**
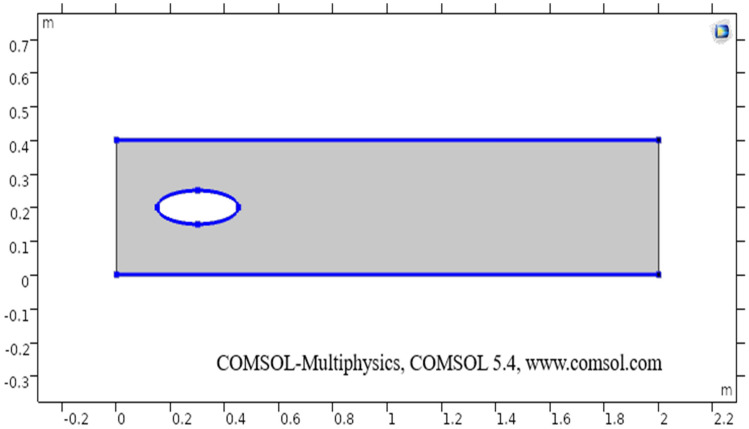
The sketch of two-dimensional elliptic cylindrical flow.

In other words the R.H.S of Eq. (3), actually represents the work done by pressure change, is the result of heat under adiabatic compression as well as some thermo-acoustic effects, it is generally low for low mac number or for compressible fluid flow. See, Tables 1, 2, and 3 for thermophysical properties, mesh statistics and mesh quality respectively. Figure 1 shows the boundary wall at which the velocity component is zero. The boundary conditions are considered and an inlet (entrance), outlet (exit) is selected in COMSOL. The Naiver-Stokes equations are handled numerically and the boundary conditions are as follow:

- “Domain” (Region)
- “Outer” (Start)

**Table 1:** The following parameters have been utilized in calculations.

Properties	Numeric value	Description
Density	997 kg/m^3^	Density of fluid
Temperature	20 °C	Temperature of fluid
Dynamic viscosity	8.90 × 10^−4^ m^2^/s	Dynamic viscosity of fluid
Specific heat ratio	1.330	Fluid’s specific heat ratio
Thermal conductivity	0.613 K(W/m K)	Thermal conductivity of fluid
Temperature 1	298 (Kelvin)	The temperature at the upper boundary
Temperature 2	300.5 (Kelvin)	The temperature at the lower boundary
Size of particle	9 nanometer (nm)	Size of the particles in the fluid

**Table 2:** Mesh statistics elaboration.

Property	Value
Minimum entity quality	0.3727
Average entity quality	0.8484
Triangular entities	23,858
Quad entities	1804
Edge entities	986
Vertex entities	8

**Table 3:** Elaboration of mesh size.

Geometric entity level	Boundary
Calibrate for	Fluid dynamics
Selection	Boundaries 2–3, 5–8
Maximum entity size	0.014
Minimum entity size	4E−4
Curvature factor	0.3
The maximum entity growth	1.13

the conditions on the lower boundary are as follow:

16 $${\text{u}}_{{\text{r}}} = {\text{u}}_{{\uptheta }} = {\text{u}}_{{\text{z}}} = 0\;{\text{and}}\;{\text{p}} = 0,\;{\text{all}}\;{\text{Natural}}\;{\text{line}}\;{\text{to}}\;\left( {0.4,0} \right)$$

no slip condition is considered at boundary I.e (u = 0)

17 $$\left\{ {\begin{array}{*{20}l} {{\text{u}}_{{\text{r}}} = {\text{u}}_{{\uptheta }} = {\text{u}}_{{\text{z}}} = 0\;{\text{and}}\;{\text{p}} = 0, \;{\text{all}}\;{\text{Natural}}\;{\text{line}}\;{\text{to}}\;\left( {0.4,2.0} \right)} \hfill \\ {{\text{u}}_{{\text{r}}} = {\text{u}}_{{\uptheta }} = {\text{u}}_{{\text{z}}} = 0\;{\text{and}}\;{\text{p}} = 0, \;{\text{all}}\;{\text{Natural}}\;{\text{line}}\;{\text{to}}\;\left( {0,2.0} \right)} \hfill \\ {{\text{u}}_{{\text{r}}} = {\text{u}}_{{\uptheta }} = {\text{u}}_{{\text{z}}} = 0\;{\text{and}}\;{\text{p}} = 0, \;{\text{all}}\;{\text{Natural}}\;{\text{line}}\;{\text{to}}\;{\text{close}}} \hfill \\ \end{array} } \right.$$

18 $$\left\{ {\begin{array}{*{20}l} {{\text{u}}_{{\text{r}}} = {\text{u}}_{{\uptheta }} = {\text{u}}_{{\text{z}}} = 0\;{\text{and}}\;{\text{p}} = 0,\;{\text{ at}}\;{\text{t}} = 0} \hfill \\ {{\text{at}}\;{\text{r}} = 0.4,\;{\text{z}} = 2.0,\;{\text{u}} = - {\text{U}}_{0} {\text{n}}.} \hfill \\ \end{array} } \right.$$

In Eq. (17), ${\text{U}}_{0}$ represents the normal flow velocity and n is the unit vector, the negative in (18) illustrate that from a higher concentration area the flow is moving towards lower concentration parts of geometry. Equation (16), basically represents the initial conditions that are under the laminar flow section. Velocity components ${\text{u}}_{{\text{r}}} ,{\text{ u}}_{{\uptheta }} ,{\text{ u}}_{{\text{z}}}$ in respective directions r, ${\uptheta }$, $z$ are chosen zero also pressure was kept zero. The slip condition is not $\left( {{\text{u}} = 0} \right)$ considered under the same section.

## Solution of the problem

Mesh is created for problem utilizing physics controlled finer option, the total number of triangular entities are 23,858, Quadrilateral entities are 1804, edge entities are 986 and the vertex entities are 8. The maximum entity size taken is 0.014 and the minimum entity size taken is 4E−4. Corner refinement in domain 1 is selected COMSOL and the minimum angle between the boundaries is 240°, sharp corners are handled through the trimming option in the COMSOL. The designed model is studied under laminar flow coupled with heat transfer in fluids feature. Finally, the designed model is computed in COMSOL and the results are obtained.

The thermophysical properties that have been utilized for laminar flow and couples heat transfer are presented in Table 1. The mesh constructed with help of COMSOL has several geometric entities. Table 2, presents all those entities which have been created along with their quality and the total number of different entities. The mesh is created for a particular section, applicable boundaries have been given, the maximum and minimum size of entities are presented in Table 3.

## Discussion and results

The present section discusses the laminar flow of viscous compressible fluid in elliptic cylindrical geometry (see) Fig. 1. The two-dimensional model for laminar flow coupled with heat transfer has been designed in COMSOL. In the two-dimensional plots, the vertical and horizontal axis present’s the diameter of the elliptic cylinder and z-direction respectively. Figure 1 describes a sketch of the drawn geometry for elliptic cylindrical flow.

**Figure 2:**
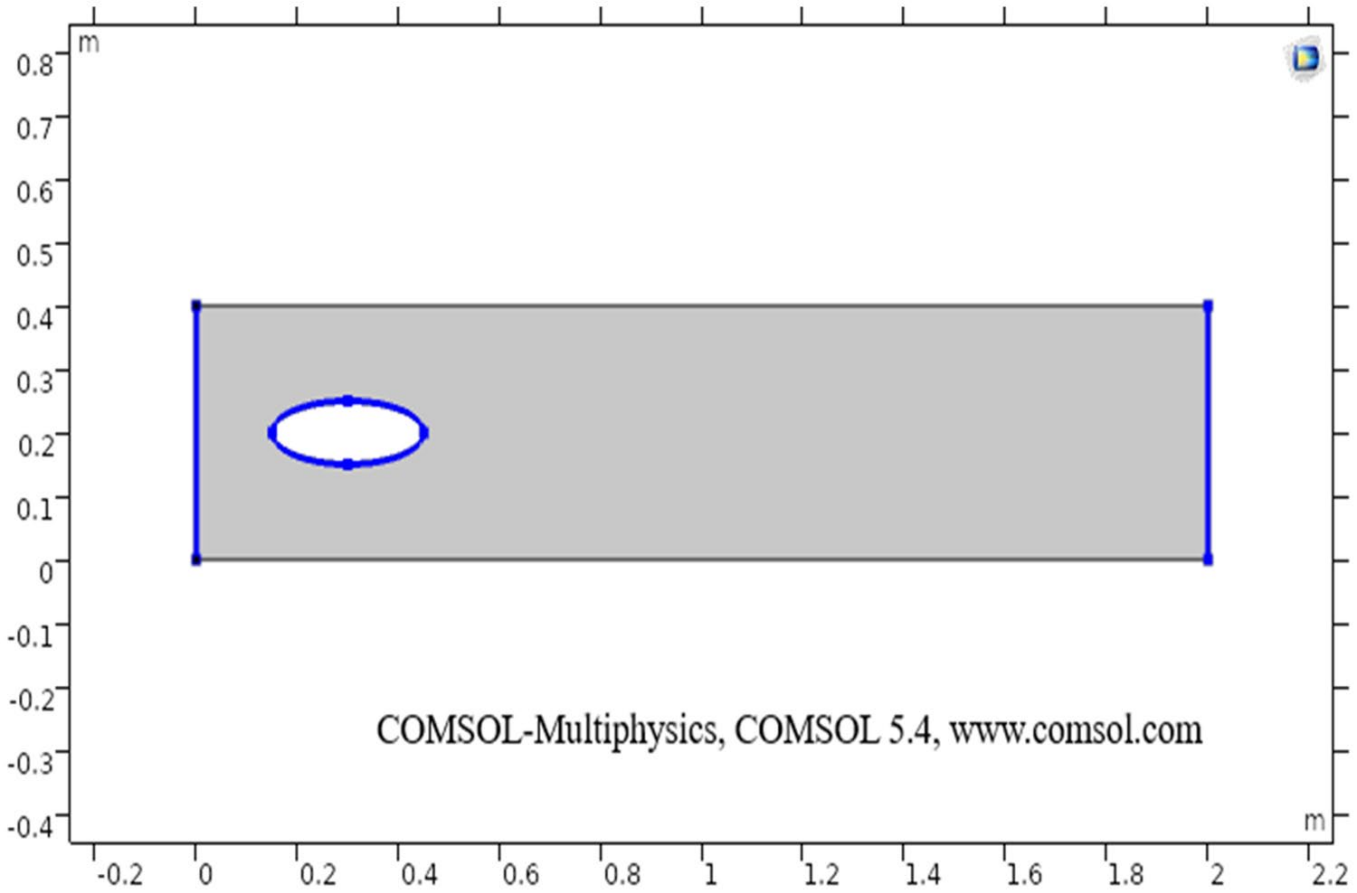
The thermally insulated boundary of flow geometry.

**Figure 3:**
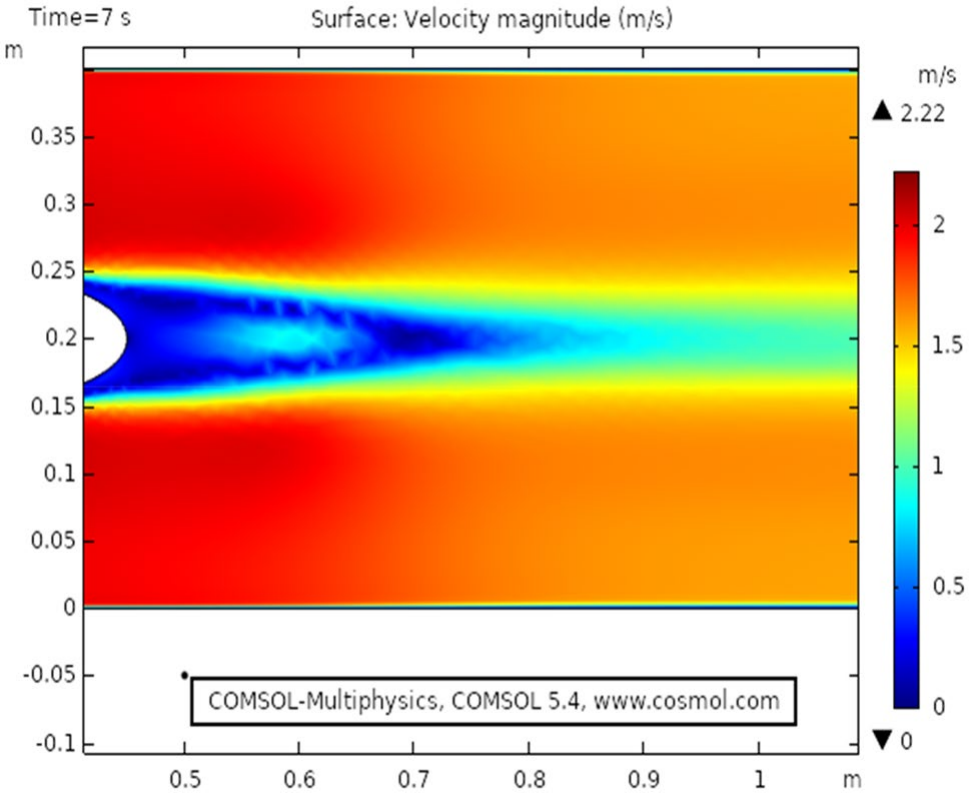
The velocity distribution in cylindrical elliptic flow at ${\text{t}} = 7\;{\text{s}}$.

**Figure 4:**
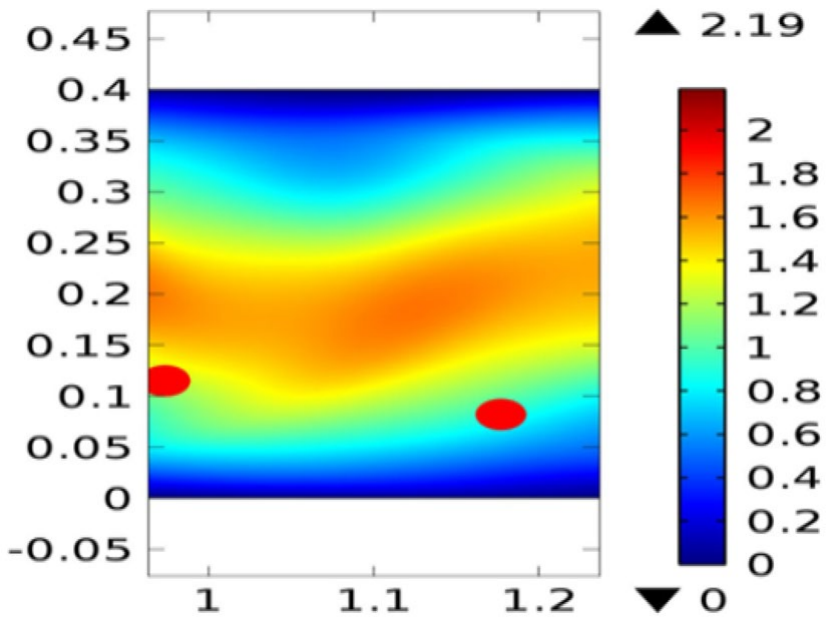
The velocity distribution in cylindrical circular flow^32^ at ${\text{t}} = 7\;{\text{s}}$.

**Figure 5:**
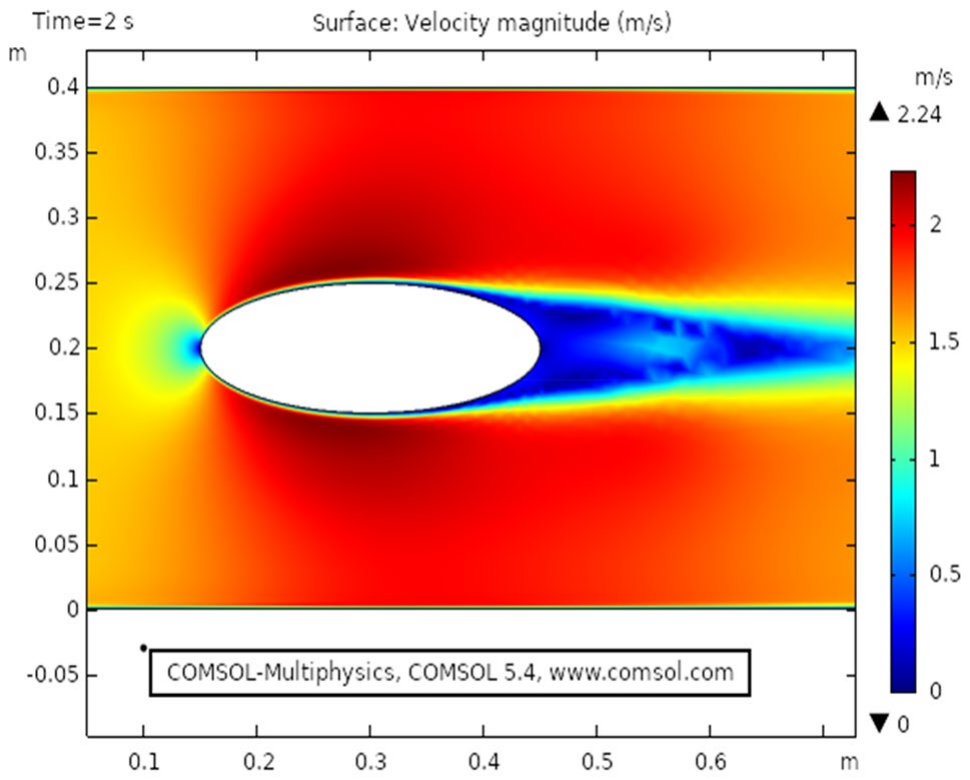
The velocity distribution in cylindrical elliptic flow at $t = 2\;{\text{s}}$.

**Figure 6:**
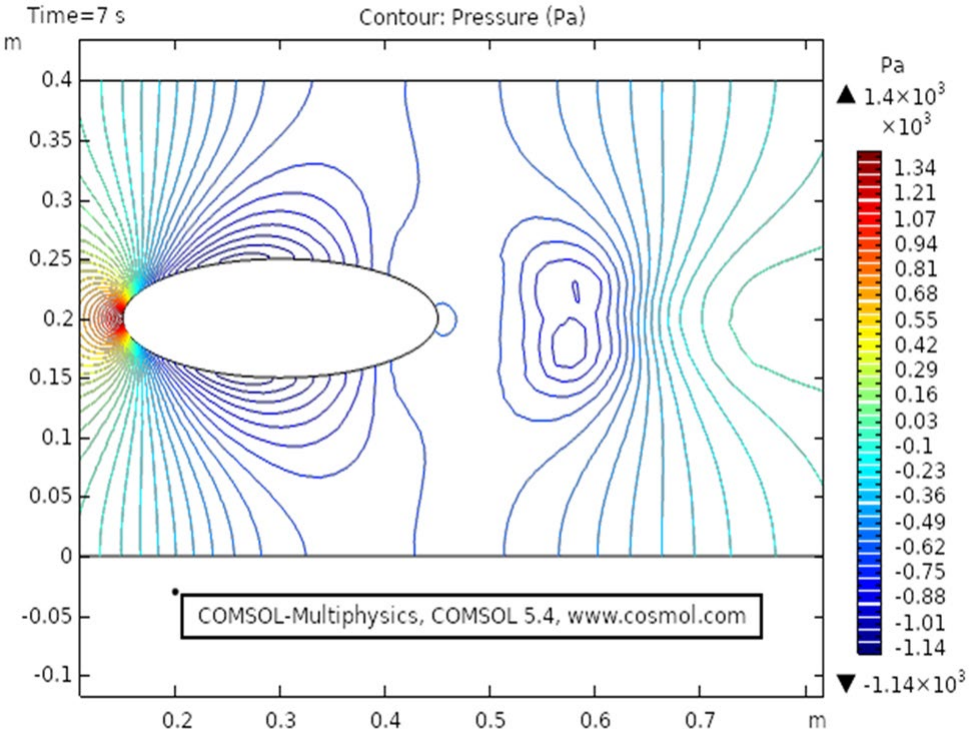
The pressure distribution in cylindrical elliptic flow at $t = 7\;{\text{s}}$.

**Figure 7:**
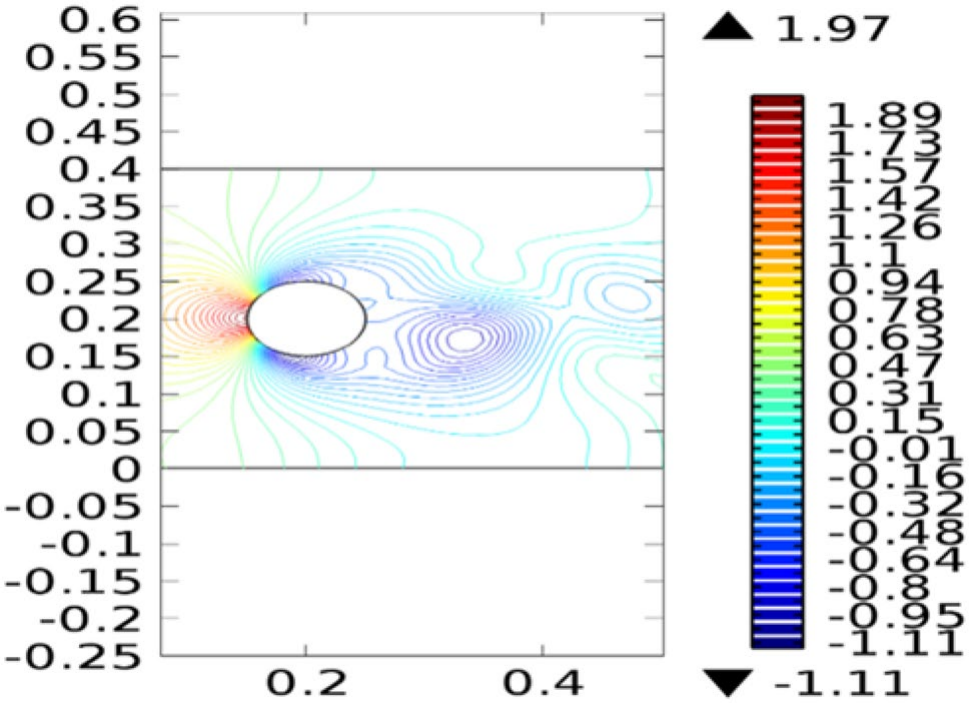
The pressure distribution in cylindrical circular flow^32^ at $t = 7\;{\text{s}}$.

**Figure 8:**
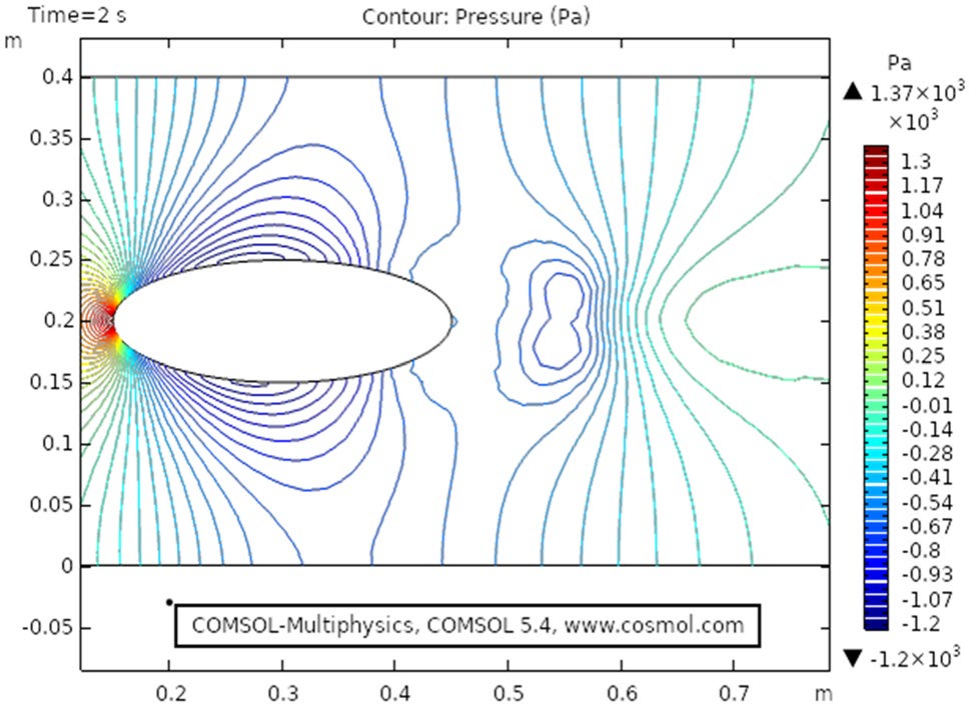
The pressure distribution in cylindrical elliptic flow at $t = 2\,{\text{s}}$.

Figure 2 shows the isothermal boundary of geometry for the heat transfer feature. Figures 3 and 4^32^ show the velocity distribution at time 7 s, as we move away from our designed geometry the velocity profile stays constant at 1.5 m/s at the center, and the velocity profile is just above, below the central part, is around 1.7 m/s. The velocity in this plot observed minimum near upper and lower boundary walls under the influence of applied temperature. The maximum observed velocity at 7 s is 2.22 m/s as shown in the figure. This happened because of the elliptic cylinder while in the circular cylinder case velocity has a maximum value of 2.19 m/s. Figure 5 describes the velocity distribution at time 2 s, it shows a distinct pattern, in this case, the maximum observed velocity is 2.24 m/s if we vary the time parameter we can even get more distinct patterns. In the region around the elliptic cylinder, the velocity is maximum while it is observed minimum at boundaries. We can see that velocity in the center part stays moderate between ${\text{u}} \ge 1$ or ${\text{u}} \le 1.5$ this suggests that if we use a circular cylinder instead of an elliptic cylinder, the velocity distribution plots can provide us even more distinct patterns. Figures 6 and 7 both illustrate pressure distribution around the elliptic cylinder, circular cylinder respectively. The distinct and clear pattern can be seen at time 7 s. This also indicates that when fluid enters the designed geometry through the inlet because of high inflow velocity the observed viscous torque is much greater as compared to any other part in geometry. The pressure distribution has a maximum value of 1.97 in the circular case (Fig. 7), as compared to the elliptic cylindrical case that is 1.89 (Fig. 6). Figure 8 shows pressure contours for time 2 s the pattern are even more clear near the boundary of the cylinder. Through this plot, it is observed that if we want to commute more satisfying and distinct patterns of pressure contours they can be achieved by using interpolation of time.

**Figure 9:**
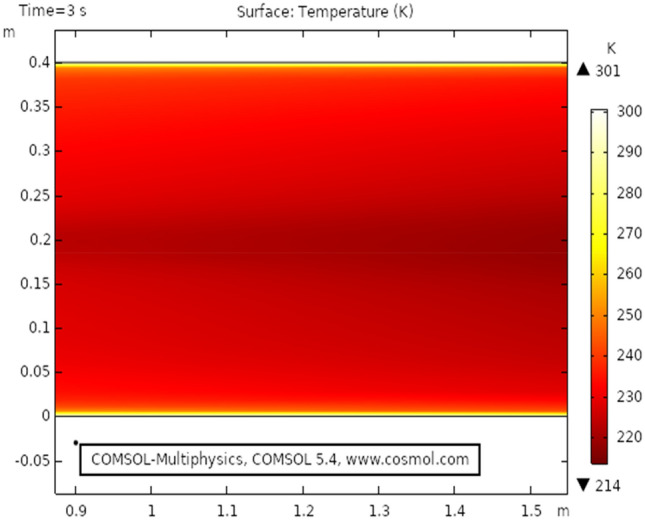
Temperature distribution at $t = 3\,{\text{s}}$.

**Figure 10:**
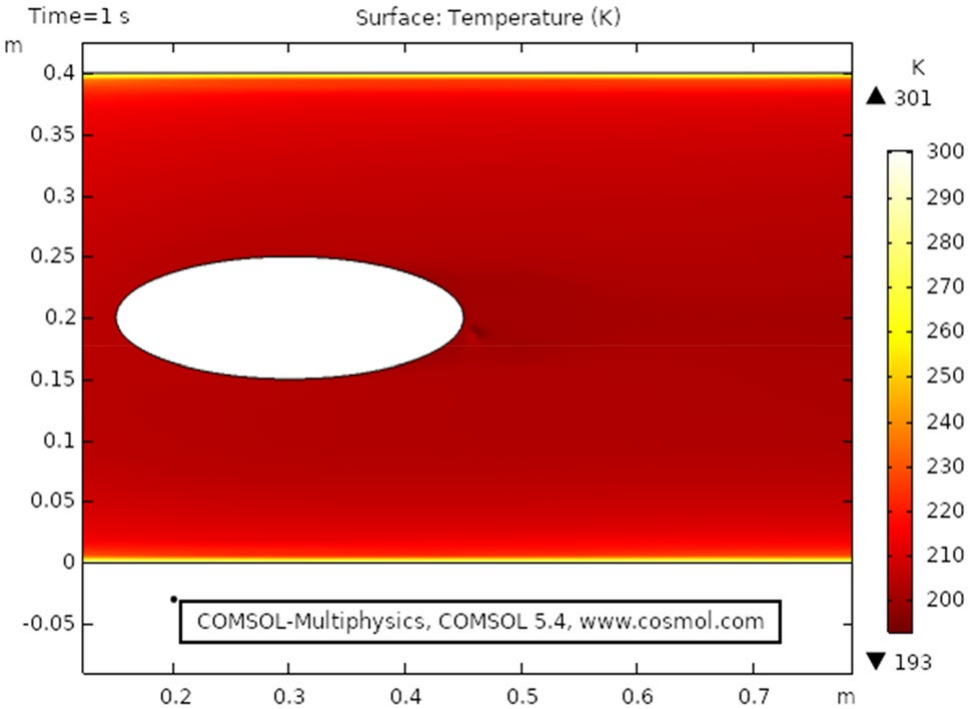
Temperature distribution at $t = 1\;{\text{s}}$.

**Figure 11:**
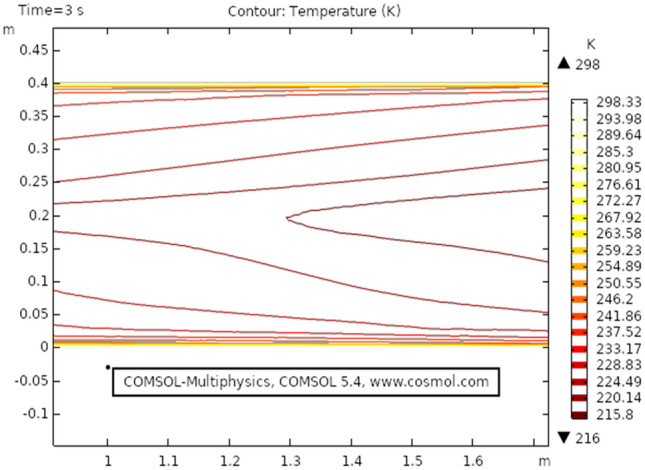
Isothermal contour temperature distribution at $t = 3\,{\text{s}}$.

**Figure 12:**
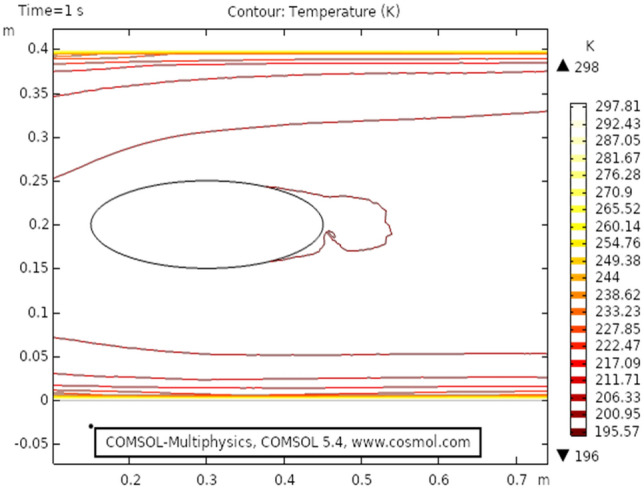
Isothermal contour temperature distribution in elliptic cylindrical flow at $t = 1\,{\text{s}}$.

Figure 9 shows the temperature profile for the heat transfer through viscous dissipation at time 3 s, it is clear from the figure that the value of temperature starts increasing as we travel from the upper boundary towards the lower boundary. The maximum temperature profile, in this case, is 301 K that can be seen in the figure. Figure 10 describes temperature distribution at time 1 s, as the result near the boundary of the elliptic cylinder temperature shift is low while at the upper and lower boundary the temperature shift is observed maximum. It is because the heat transfer through viscous fluid has just been initiated, as time passes a gradual pattern of heat transfer can be observed. Figure 11 the isothermal contours of temperature are presented at time 3 s, a unique pattern is obtained at this stage value of temperature profile has slightly decreased as observed from the figure. In this case, the maximum value of the temperature shift is 299 K. Figure 12 shows the isothermal contours at time 1 s, the pattern is moving away from the elliptic cylinder as shown in the figure, in this case, the maximum temperature observed is 298 K. It can be observed that near the lower boundary the pattern overlaps because of high-temperature distribution.

**Figure 13:**
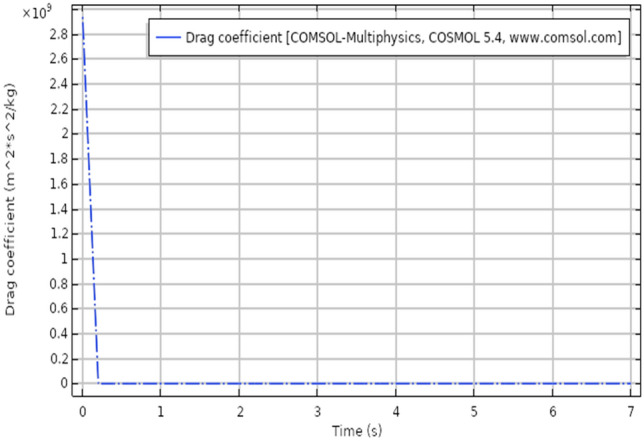
Drag coefficient for laminar flow.

**Figure 14:**
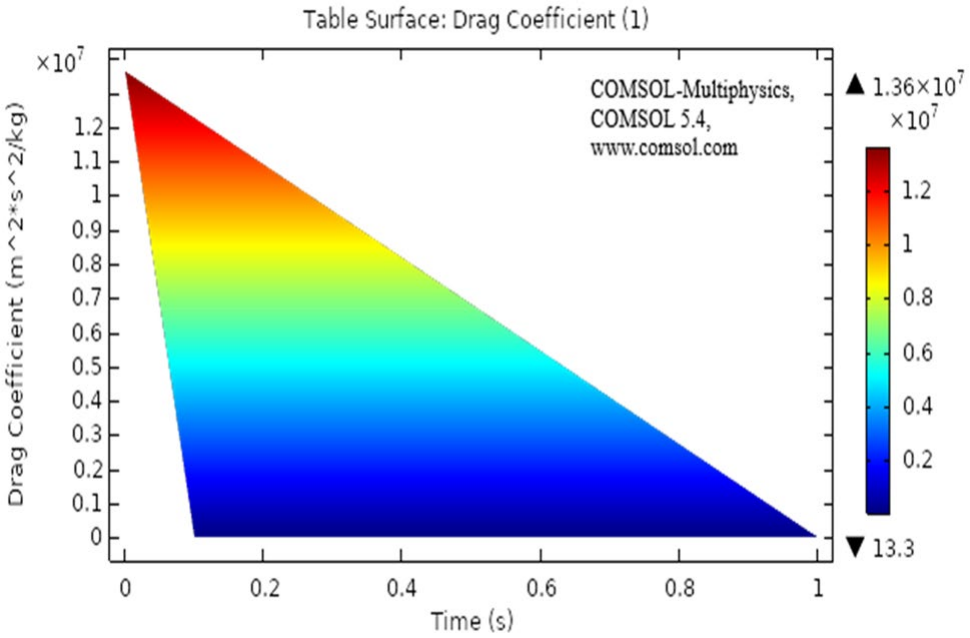
Surface table of drag coefficient for laminar flow.

The drag force acts parallel to flow direction and lift acts perpendicular to flow direction, the lift and drag coefficient has been discussed in^32^ similarly under the regime of magnetic field Fig. 13 shows drag coefficient behavior for the present problem, the drag force shows a sudden decrease when the time scale reaches 0.5 s, afterward the drag force shows constant linear behavior. The maximum drag was observed near the inlet and a sudden drop is caused after the laminar flow has passed the elliptic cylinder. Figure 14 describes the surface change drag coefficient, the drag force is maximum at time 0 s as seen from the table. The maximum value for drag is 1.36. It also describes that how the drag intensity decreases as the elliptic cylinder is crossed by fluid.

## Concluding remarks

Present results are discussed under the heat transfer effects. the model is time-dependent. The model for the present study has been modeled in COMSOL, the viscous compressible flow has shown a distinct pattern for relevant parameters. It is clear from the results that heat dissipation effect's the velocity of viscous fluid. This particular simulation can play a vital role in applications like forced air-furnaces and automobile cooling systems. The airflow across the heat radiator can also be benefited.

- The velocity distribution with heat transfer show varying distinctively for time-dependent laminar flow. The maximum velocity observed is 2.22 m/s.
- The pressure distribution profile has also shown a clear pattern but as we move away elliptic cylinder the pattern disappear because of varying time. The pressure distribution has a maximum value of 1.89 Pa.s.
- The isothermal contours are very clear and distinct for each step of the time. The contours vary with varying time, the maximum value of temperature is 299 K.
- The drag coefficient has shown linear behavior after a very slight change of time. The drag force was observed to decrease and showed a sudden drop with minimal time change.

## List of symbols

$${\text{F}}_{{\text{b}}}$$: Body forces
${\uprho }$: Density of fluid
${\text{d}}_{{\text{z}}}$: The thickness of designed geometry
${\text{c}}_{{\text{p}}}$: Constant pressure heat capacity
$\nabla {\text{T}}$: The gradient of absolute temperature
${\text{q}}$: Heat flux quantity
${\text{Q}}$: Heat source other than viscous dissipation
${\text{q}}_{0}$: Heat flux at a certain time
${\text{Q}}_{{\text{p}}}$: Heat dissipation at constant pressure
${\text{Q}}_{{\text{vd }}}$: Viscous heat dissipation in the fluid
$\uptau$: Viscous stress tensor for fluid
$$\Delta {\text{T}}$$: Temperature difference
${\text{A}}_{{\text{s}}}$: Area of geometry
t: Time
$${\text{Pr}}$$: Prandtl number
$${\upeta }$$: Dimensionless parameter
$${\upnu }$$: Dynamic viscosity
$${\upmu }$$: Kinematic viscosity
$${\uptheta }$$: Dimensionless temperature
$${\text{k}}$$: Thermal conductivity
K: Temperature in Kelvin
${\text{u}}_{{\text{r}}}$, ${\text{u}}_{{\uptheta }}$, ${\text{ u}}_{{\text{z}}}$: Velocity components in r, θ, and z
$${\text{T}}_{{\text{w}}}$$: Wall temperature
${\text{T}}_{\infty }$: Free stream velocity
T: Absolute temperature
${\text{I}}$: Identity matrix of 3 × 3
${\text{U}}_{0}$: Normal inflow velocity
Pa: Pascal (unit for pressure)

## References

[1] Nadeem S, Khan MN, Muhammad N, Ahmad S (2019). Mathematical analysis of bio-convective micropolar nanofluid. J. Comput. Des. Eng..

[2] Nadeem S, Abbas N, Khan AU (2018). Characteristics of three-dimensional stagnation point flow of a hybrid nanofluid past a circular cylinder. Results Phys..

[3] Nadeem S, Rehman A, Lee C, Lee J (2012). Boundary layer flow of second grade fluid in a cylinder with heat transfer. Math. Probl. Eng..

[4] Turkyilmazoglu M (2009). Purely analytic solutions of the compressible boundary layer flow due to a porous rotating disk with heat transfer. Phys. Fluids.

[5] Asghar S, Jalil M, Hussan M, Turkyilmazoglu M (2014). Lie group analysis of flow and heat transfer over a stretching rotating disk. Int. J. Heat Mass Transf..

[6] Turkyilmazoglu M (2012). Effects of uniform radial electric field on the MHD heat and fluid flow due to a rotating disk. Int. J. Eng. Sci..

[7] Sajid MU, Ali HM (2018). Thermal conductivity of hybrid nanofluids: A critical review. Int. J. Heat Mass Transf..

[8] Rehman A, Hussain A, Nadeem S (2021). Physical aspects of convective and radiative molecular theory of liquid originated nanofluid flow in the existence of variable properties. Phys. Scr..

[9] Hussain A, Rehman A, Nadeem S, Malik MY, Issakhov A, Sarwar L, Hussain S (2021). A combined convection Carreau–Yasuda nanofluid model over a convective heated surface near a stagnation point: A numerical study. Math. Probl. Eng..

[10] Mebarek-Oudina F, Redouane F, Rajashekhar C (2020). Convection heat transfer of MgO-Ag/water magneto-hybrid nanoliquid flow into a special porous enclosure. Alger. J. Renew. Energy Sustain. Dev..

[11] Rehman A, Hussain A, Nadeem S (2021). Assisting and opposing stagnation point pseudoplastic nano liquid flow towards a flexible Riga sheet: A computational approach. Math. Probl. Eng..

[12] Hussain A, Alshbool MH, Abdussattar A, Rehman A, Ahmad H, Nofal TA, Khan MR (2021). A computational model for hybrid nanofluid flow on a rotating surface in the existence of the convective condition. Case Stud. Thermal Eng..

[13] Abo-Dahab SM, Abdelhafez MA, Mebarek-Oudina F, Bilal SM (2021). MHD Casson nanofluid flow over nonlinearly heated porous medium in presence of extending surface effect with suction/injection. Indian J. Phys..

[14] Mebarek-Oudina F (2019). Convective heat transfer of Titania nanofluids of different base fluids in the cylindrical annulus with the discrete heat source. Heat Transf.—Asian Res..

[15] Swain K, Mebarek-Oudina F, Abo-Dahab SM (2021). Influence of MWCNT/Fe_3_O_4_ hybrid nanoparticles on an exponentially porous shrinking sheet with chemical reaction and slip boundary conditions. J. Therm. Anal. Calorim..

[16] Van Driest ER (1952). Calculation of the stability of the laminar boundary layer in a compressible fluid on a flat plate with heat transfer. J. Aeronaut. Sci..

[17] Vajravelu K (2001). Viscous flow over a nonlinearly stretching sheet. Appl. Math. Comput..

[18] Lin A, Rubin SG (1982). Three-dimensional supersonic viscous flow over a cone at incidence. AIAA J..

[19] Malik MR, Spall RE (1991). On the stability of compressible flow past axisymmetric bodies. J. Fluid Mech..

[20] Cantwell BJ (1996). Fundamentals of Compressible Flow.

[21] Kumar BR, Sivaraj R (2013). Heat and mass transfer in MHD viscoelastic fluid flow over a vertical cone and flat plate with variable viscosity. Int. J. Heat Mass Transf..

[22] Houda S, Belarbi R, Zemmouri N (2017). A CFD Comsol model for simulating complex urban flow. Energy Proc..

[23] Fröhlingsdorf W, Unger H (1999). Numerical investigations of the compressible flow and the energy separation in the Ranque–Hilsch vortex tube. Int. J. Heat Mass Transf..

[24] Yang W, Lee KK, Choi S (2017). A laminar-flow-based microbial fuel cell array. Sens. Actuators, B Chem..

[25] Barnoon P, Toghraie D (2018). Numerical investigation of laminar flow and heat transfer of non-Newtonian nanofluid within a porous medium. Powder Technol..

[26] Ahmad, M. F., Haniffah, M. R. M., Kueh, A. & Kasiman, E. H. Numerical study on drag and lift coefficients of a marine riser at high Reynolds number using COMSOL multiphysics. In *IOP Conference Series: Earth and Environmental Science*, Vol. 476, No. 1, 012075 (IOP Publishing, 2020).

[27] Zhou X, Yang J, Xiao B, Hou G, Wu Y (2009). Numerical investigation of a compressible flow through a solar chimney. Heat Transf. Eng..

[28] Rehman FU, Nadeem S, Haq RU (2017). Heat transfer analysis for three-dimensional stagnation-point flow over an exponentially stretching surface. Chin. J. Phys..

[29] Hussain A, Ullah A (2016). Boundary layer flow of Walter's B fluid due to a stretching cylinder with temperature-dependent viscosity. Alex. Eng. J..

[30] Naseer M, Malik MY, Nadeem S, Rehman A (2014). The boundary layer flow of hyperbolic tangent fluid over a vertical exponentially stretching cylinder. Alex. Eng. J..

[31] Hussain A, Sarwar L, Akbar S, Nadeem S, Jamal S (2019). Numerical investigation of viscoelastic nanofluid flow with radiation effects. Proc. Inst. Mech. Eng., Part N: J. Nanomater., Nanoeng., Nanosyst..

[32] Bhandari A (2021). Numerical study of time-dependent ferrofluid flow past a cylinder in the presence of the stationary magnetic field. SN Appl. Sci..

[33] Saleem S, Nadeem S, Sandeep N (2017). A mathematical analysis of time-dependent flow on a rotating cone in a rheological fluid. Propuls. Power Res..

